# Cellular polyamines condense hyperphosphorylated Tau, triggering Alzheimer’s disease

**DOI:** 10.1038/s41598-020-67119-x

**Published:** 2020-06-22

**Authors:** Stefan M. Ivanov, Mariyana Atanasova, Ivan Dimitrov, Irini A. Doytchinova

**Affiliations:** 1 Faculty of Pharmacy, Medical University of Sofia, Dunav 2 st., Sofia, 1000 Bulgaria; 2 Present Address: Institute for Bioscience and Biotechnology Research, University of Maryland, Rockville, MD USA

**Keywords:** Computational biophysics, Intrinsically disordered proteins, Kinetics, Molecular conformation, Supramolecular assembly, Thermodynamics, Mechanisms of disease, Alzheimer's disease, Computational chemistry, Molecular dynamics, Statistical mechanics

## Abstract

Many gaps in our understanding of Alzheimer’s disease remain despite intense research efforts. One such prominent gap is the mechanism of Tau condensation and fibrillization. One viewpoint is that positively charged Tau is condensed by cytosolic polyanions. However, this hypothesis is likely based on an overestimation of the abundance and stability of cytosolic polyanions and an underestimation of crucial intracellular constituents – the cationic polyamines. Here, we propose an alternative mechanism grounded in cellular biology. We describe extensive molecular dynamics simulations and analysis on physiologically relevant model systems, which suggest that it is not positively charged, unmodified Tau that is condensed by cytosolic polyanions but negatively charged, hyperphosphorylated Tau that is condensed by cytosolic polycations. Our work has broad implications for anti-Alzheimer’s research and drug development and the broader field of tauopathies in general, potentially paving the way to future etiologic therapies.

## Introduction

Alzheimer’s disease (AD) is a progressive neurodegenerative disease characterized by the buildup of amyloid plaques in the extracellular space of nerve tissue^1^ and the impairment of activities of daily living^2^. The increase in life expectancy and morbidity of Alzheimer’s disease inevitably lead to an increased socioeconomic burden on developed and developing nations^3^. Thus, there is an urgent need of a pharmacotherapeutic solution. Up until now, the main molecular target in anti-Alzheimer’s drug development^4^ has been amyloid beta (Aβ) – the main component of amyloid senile plaques found in the brains of Alzheimer’s patients^5^. The discontinuation of all aducanumab^6^ clinical trials – the latest in a long line of unsuccessful antiamyloid drugs^7,8^ – has called into question the amyloid hypothesis and its main corollary – that Aβ should be the primary target of anti-Alzheimer’s drug development. The string of failures has prompted the research community to look into other potential targets^9^. One promising target is the microtubule-binding protein Tau – the main constituent of neurofibrillary tangles^10^ (NFTs). NFTs, like amyloid plaques, are a hallmark of Alzheimer’s disease. However, unlike amyloid plaques, they are intracellular, being overwhelmingly localized inside neurons^11^. Although symptoms correlate strongly with NFT prevalence^12,13^, much more so than with amyloid senile plaques^14,15^, little is known and understood about their origin. Tau has several isoforms which differ in their propensity to form tangles^16^. Some of the most tangle-prone of these are the three microtubule repeat-binding and four microtubule repeat-binding (3R- and 4R-Tau, respectively). Moreover, it has been shown that neurofibrillary tangles appear only after hyperphosphorylation, where Tau comes to bear 10 or more moles of phosphate per mole of protein, as opposed to the physiological norm of 2–3 moles^17–19^. This appears counterintuitive, as most Tau isoforms, especially its tangle-forming ones, are low in hydrophobic residues, intrinsically disordered, and highly soluble. Moreover, introducing 10 moles of phosphate in 3R- and 4R-Tau gives them a net negative charge of around −15 and −10, respectively, suggesting a propensity for selfrepulsion, rather than selfattraction and aggregation. Recent *in vitro* experiments have shown that unmodified 3R- and 4R-Tau undergo concentration-dependent liquid-liquid phase separation (LLPS) at a concentration of around 100 μM for 4 R, the more aggregation-prone of the two isoforms^20^. This is likely due to the presence of large numbers of positive (21 lysines and 1 arginine) and negative (7 glutamic and 3 aspartic acid) residues present in the 4R-Tau sequence that was studied. Moreover, it was found that phosphorylated 4R-Tau condenses at much lower concentrations (~2 μM). Crucially, it was shown that in the presence of negative polyions, such as heparin and, potentially, RNA, condensed, unmodified 3R- and 4R-Tau rapidly undergo an additional phase transition, forming solid-like amyloid fibrils from an initial liquid phase^20^. Thus, a picture of lysine-rich, positively charged Tau being condensed by negatively charged RNAs and heparin emerges. However, the aforementioned study does not account for several crucial circumstances of cellular biology. First, heparin and the much larger class of glycosaminoglycans in general are overwhelmingly extracellular substances^21–23^; what little of them is present inside cells is sequestered from the cytosol in the granules of mast cells, ready for secretion, or in endosomes and lysosomes, ready for degradation^24^. Second, most RNAs have very short half-lives, on the order of minutes for most messenger RNAs^25^, with the vast majority of longer-lived RNAs sequestered in nucleoli, ribosomes, and stress granules^26^. Thus, it seems doubtful whether the heparin-induced Tau fibrillization observed *in vitro* and the proposed RNA-induced fibrillization are physiologically relevant, i.e. occur *in vivo* to any significant degree. Lastly, the aforementioned study, as well as similar studies before and after it, do not take into account the presence of polyamines inside cells – polycations that are constitutively present inside the nucleus and cytosol and perform multiple essential physiological functions^27–30^. Crucially, the main cellular polyamines in vertebrates^31^ - spermine, with a net charge of +4, and spermidine, with a net charge of +3 - have been shown to condense DNA *in vitro* and *in vivo*^32,33^. Here, we present extensive molecular dynamics simulations and analysis, grounded in cellular biology, which challenge the *in vitro* view, expressed by certain researchers, of positively charged Tau being condensed into neurofibrillary tangles by negatively charged heparin and RNA, and supplant it with a physiologically relevant picture of negatively charged hyperphosphorylated Tau being condensed by positively charged cellular polyamines – spermine and spermidine. Our results offer insight into the atomistic mechanisms of neurofibrillary tangle formation in Alzheimer’s disease and the broader class of tauopathy^34^ in general, and potentially open the way to developing etiologic treatment and prevention in the future, as opposed to the symptomatic pharmacotherapeutic strategies of the present time.

## Results

### Spermine is repulsed by unmodified 4R-Tau and attracted to the hyperphosphorylated variant

In order to assess the behavior of the cellular polyamine spermine in the presence of unmodified and hyperphosphorylated 4R-Tau (henceforth also referred to simply as Tau), we performed 1 μs-long all-atom molecular dynamics (MD) simulations of both variants in the presence of spermine, placing 8 Tau chains and 44 spermine molecules in the unit cell, along with a physiological concentration of NaCl. In all of our subsequent analyses, we used frames taken 100 ps (0.1 ns) apart during the molecular dynamics trajectories; this afforded ample, fine-grained data to base our conclusions on.

**Figure 1:**
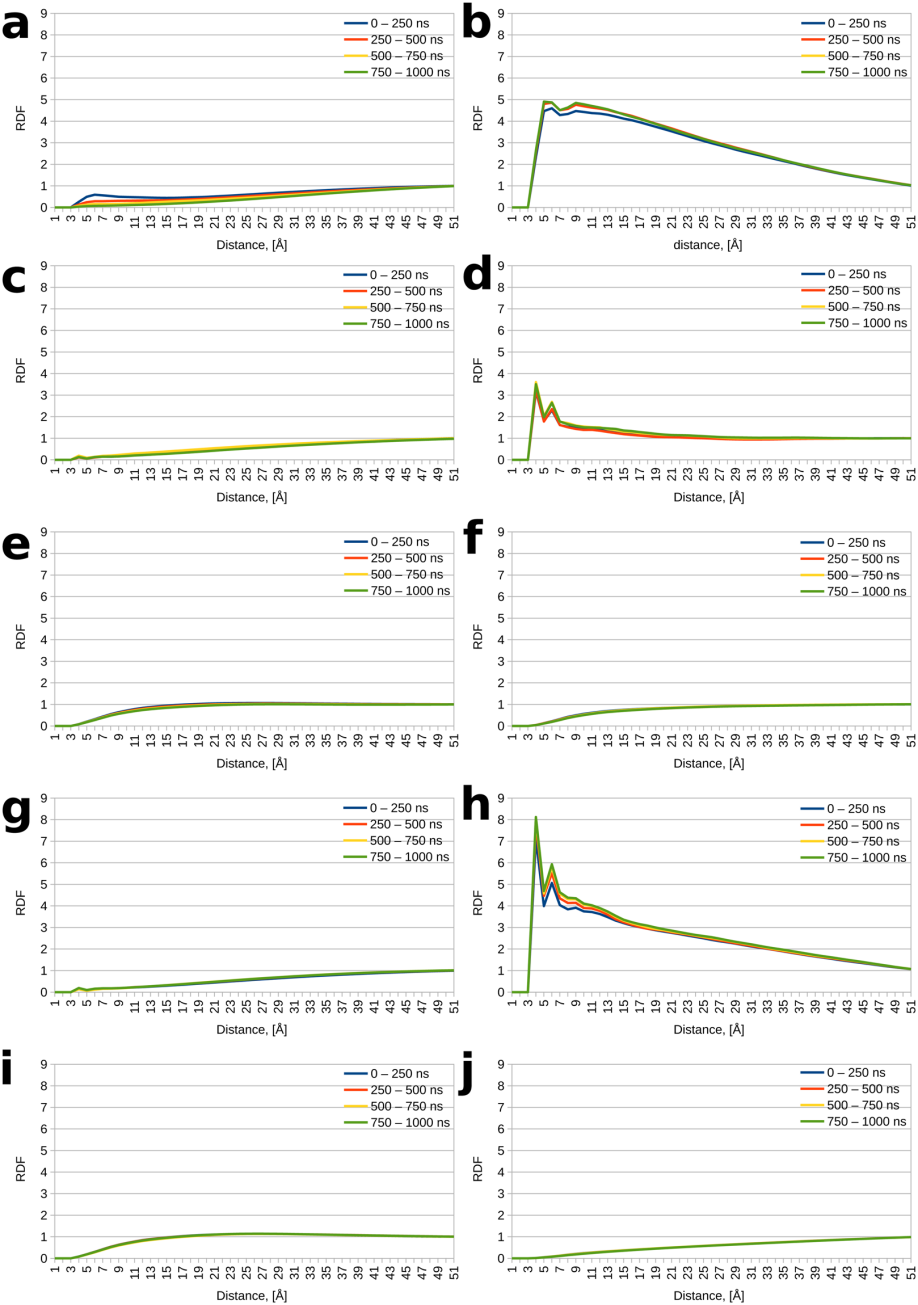
Radial distribution functions (RDFs) for the different moieties around the protein. (**a**) RDF values for spermine around unmodified Tau. (**b**) RDF values for spermine around phosphorylated Tau. (**c**) RDF values for Na^+^ around unmodified Tau (spermine is present in the system). (**d**) RDF values for Na^+^ around phosphorylated Tau (spermine is present in the system). (**e**) RDF values for Cl^−^ around unmodified Tau (spermine is present in the system). (**f**) RDF values for Cl^−^ around phosphorylated Tau (spermine is present in the system). (**g**) RDF values for Na^+^ around unmodified Tau (spermine is not present in the system). (**h**) RDF values for Na^+^ around phosphorylated Tau (spermine is not present in the system). (**i**) RDF values for Cl^−^ around unmodified Tau (spermine is not present in the system). (**j**) RDF values for Cl^−^ around phosphorylated Tau (spermine is not present in the system).

We first analyzed the radial distribution function (RDF) of spermine heavy atoms around protein heavy atoms, splitting the microsecond-long trajectories in four 250 ns-long blocks to assess the convergence of the spermine distribution around the protein. The radial distribution function affords an assessment of how likely it is for a given molecule or chemical group to be found at a given distance from another given group or molecule, as opposed to being found in bulk solvent, where the RDF converges to unity. RDF values below 1 indicate that a given molecule is less likely to be found around another given molecule at a given distance compared to being found in bulk solvent; RDF values above 1 indicate the opposite. Examining the radial distribution function of spermine around Tau reveals that spermine is less likely to be found around unmodified Tau as opposed to bulk; the opposite is true for phosphorylated Tau. Indeed, in the unmodified system, the RDFs are well below 1 in proximity to the protein and converge to unity only in bulk solvent (Fig. 1a). However, the RDFs for the four 250 ns blocks in the phosphorylated system exhibit peaks well above unity near the protein (Fig. 1b). Moreover, a closer inspection of the RDFs reveals that the curves for the 500–750 and 750–1000 ns time blocks lie close to each other. In turn, the 250–500 ns curve lies between these two curves and the 0–250 ns curve for the unmodified system; in the case of hyperphosphorylated Tau, the 250–500, 500–750, and 750–1000 ns curves are practically indistinguishable. This behavior indicates that the 1 μs simulations are sufficiently long for the spermine distribution to converge, at least to some local minimum in conformational hyperspace. For hyperphosphorylated Tau, this minimum lies in proximity to the protein, for unphosphorylated Tau – far removed from the protein in bulk solvent.

We then inspected the distribution of Na and Cl ions around the protein. Na^+^ appears to behave similarly to spermine, positioning itself away from unmodified Tau in bulk solvent and in proximity to phosphorylated Tau (Fig. 1c,d). Perhaps somewhat surprisingly, Cl ions are nonselective, preferring to position themselves in bulk solvent, regardless of the phosphorylation state of Tau (Fig. 1e,f), despite the abundance of positive residues (21 lysines and 1 arginine for each Tau chain) and the high net positive charge of unmodified Tau (+12). Generally, the RDF curves for the Na and Cl ions exhibit very similar behavior over the four time blocks. This suggests that their distribution has mostly converged within the first 250 ns of production dynamics, which is to be expected, given that they are much smaller and more mobile than spermine.

We also examined the behavior of Na^+^ and Cl^−^ in analogous systems not containing spermine (see Fig. 1g-j), as this allows us to specifically assess the influence of the polyamine and its effects on the behavior of the other system components. It was found that the ions exhibit very similar behavior in terms of RDF curves and convergence to the spermine-containing systems, the only notable differences being the longer time needed for the Na^+^ distribution to converge and the higher peaks for Na^+^ around phosphorylated Tau in the system without spermine compared to the one with spermine (compare Fig. 1h to Fig. 1d). The first difference is likely due to the greater number of sodium ions in the system without spermine (238 vs 150); the second difference suggests that spermine actively competes with Na^+^ for protein binding sites.

### Phosphorylation enhances intermolecular Tau – Tau bridging interactions

**Figure 2:**
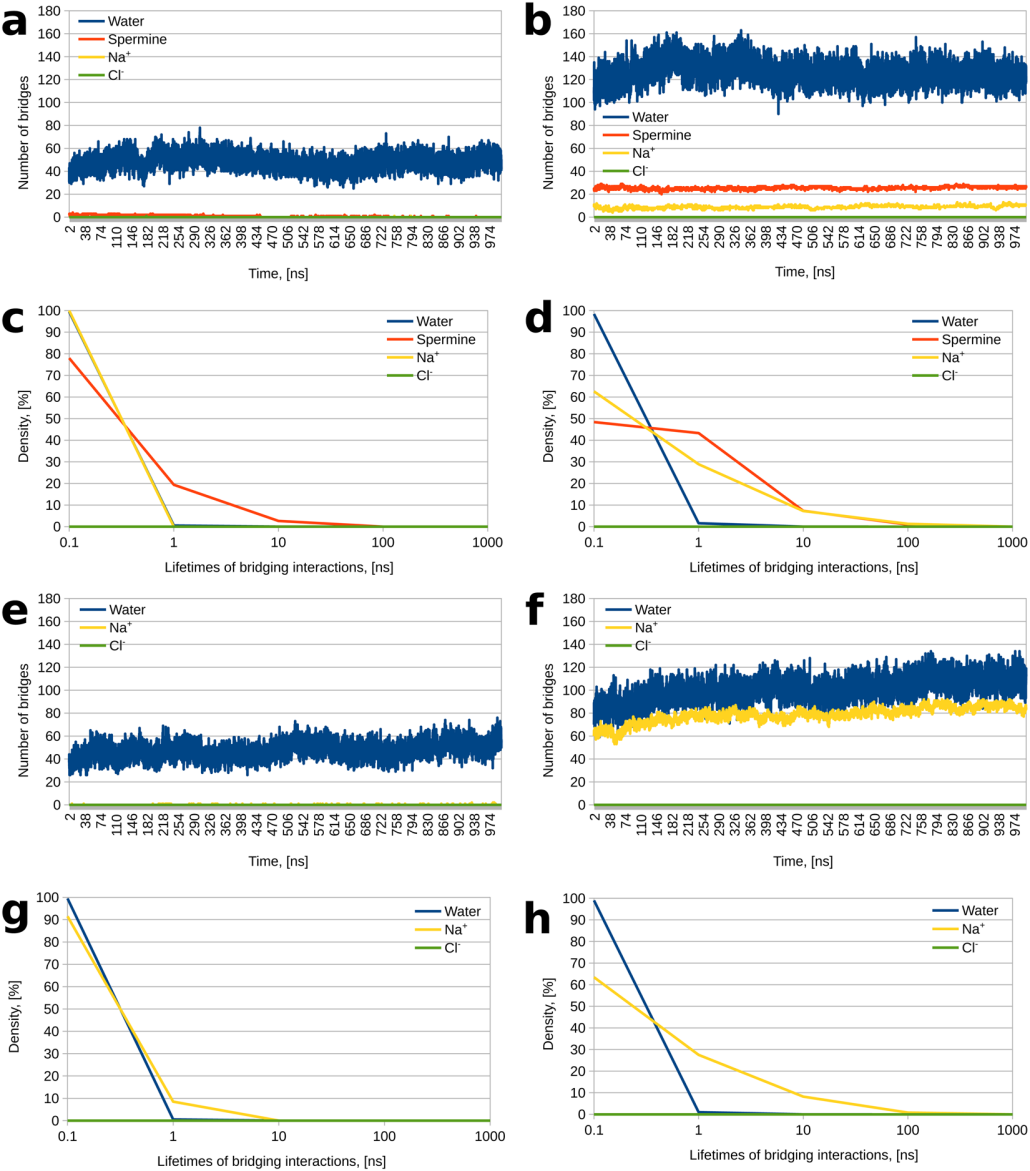
Number and distribution of bridging interactions. (**a**) Number of water-, spermine-, and ion-mediated bridging interactions as a function of time in the unmodified Tau system. (**b**) Number of water-, spermine-, and ion-mediated bridging interactions as a function of time in the phosphorylated Tau system. (**c**) Distribution of water-, spermine-, and ion-mediated bridging interactions by lifetime (i.e., the duration of the bridging interactions) in the unmodified Tau system. Note that the blue and yellow curves largely overlap and are hard to distinguish. (**d**) Distribution of water-, spermine-, and ion-mediated bridging interactions by lifetime (i.e., the duration of the bridging interactions) in the phosphorylated Tau system. (**e**) Number of water- and ion-mediated bridging interactions as a function of time in the unmodified Tau system without spermine. (**f**) Number of water- and ion-mediated bridging interactions as a function of time in the phosphorylated Tau system without spermine. (**g**) Distribution of water- and ion-mediated bridging interactions by lifetime (i.e., the duration of the bridging interactions) in the unmodified Tau system without spermine. (**h**) Distribution of water- and ion-mediated bridging interactions by lifetime (i.e., the duration of the bridging interactions) in the phosphorylated Tau system without spermine.

We next examined the influence of phosphorylation on the propensity of Tau to form bridging interactions between different protein chains. Here, an intermolecular bridging interaction is defined as one where an ion, water molecule or spermine molecule is simultaneously hydrogen bonded to residues belonging to different Tau chains. Our results demonstrate that phosphorylation greatly enhances the propensity of the solvent and the dissolved ions and spermine to mediate Tau – Tau interactions. In the case of unmodified Tau, around 47,000 individual water molecules participate in at least one interchain bridging interaction, with an average of around 50 bridging water interactions being present at each point throughout the molecular dynamics simulation (Fig. 2a). Moreover, there are an additional 24 different spermine molecules bridging Tau – Tau interactions, with the majority of the bridging interactions occurring near the beginning of the simulation due to the random placement of the molecules; over time, the number of spermine-mediated bridging interactions goes from 2–3 per frame to 0, as spermine moves away from the protein (Fig. 2a). Finally, for unmodified Tau, the number of ion-mediated bridging interactions is negligible (Fig. 2a). All of this is starkly contrasted by the behavior of the phosphorylated system. Here, the average number of bridging water molecules per frame increases to around 130, bridging Na^+^ and spermine also greatly increase (Fig. 2b). Notably, no bridging Cl^−^ are found in either system, despite the fact that the sequence under study is rich in positive charge (21 lysines and 1 arginine).

Further detail is added to the picture by examining the lifetimes of the bridging interactions. Plotting the density of the bridging interactions against their duration on a semi-log graph shows that phosphorylation increases not only the number but also the duration of spermine- and Na^+^-mediated interactions. In the unmodified Tau – spermine system, we observed 418 bridging interactions mediated by spermine and only 2 bridging interactions mediated by Na^+^; these were carried out by 24 different spermine molecules and 2 different sodium ions, respectively. All or 100% of the Na^+^-mediated bridging interactions had a lifetime below 1 ns (the lifetimes of the two bridging interactions were 0.1 and 0.2 ns), whereas the spermine-mediated interactions tended to be longer-lived – nearly 78% were between 0.1 and 1 ns, 19.3% were between 1 and 10 ns, with the remaining 11 interactions (out of a total of 418) existing between 10 and 100 ns (Fig. 2c, note that the blue (water) and yellow (Na^+^) curves in Fig. 2c largely overlap and are hard to distinguish); the longest bridging interaction in the system was 99.3 ns in duration. The phosphorylated Tau – spermine system, in contrast, exhibited much more numerous spermine- and Na^+^-mediated bridging interactions, which tended to be longer-lived. Here, the number of spermine-mediated bridges rose to 3,173 (compared to 418 for unmodified Tau), with multiple bridges exceeding hundreds of nanoseconds in duration, and 4 of the bridges reaching 999.9 ns in duration (note that the proportion of longer-lived interactions is higher for the red curve in Fig. 2d compared to Fig. 2c). Moreover, the number of individual spermine molecules involved in bridging interactions rises from 24 in the unmodified system to 39 in phosphorylated Tau (out of a total of 44 for both systems). Na^+^-mediated bridges exhibit a similar trend – the number of individual bridging ions increases (from 2 in unmodified Tau to 96 in phosphorylated Tau), as does the number of bridging interactions – from 2 to 1382 – with longer-lived bridges being much more prevalent (the yellow curve in Fig. 2d reaches much larger values compared to Fig. 2c). Indeed, here we observed 82 bridges between 10 and 100 ns in duration and 18 bridges between 100 and 1000 ns; the longest-lived Na^+^-mediated bridge was present for 831 ns of the 1,000 ns simulation time. Of the remaining 1,282 bridges, 970 were 0.2 ns or longer. All of this compares with just 2 bridges in the unmodified Tau – spermine system, which were of 0.1 and 0.2 ns duration. In both systems, no bridging Cl ions were observed. Notably, the distribution of bridging water molecules was practically identical in the two systems (compare the blue lines in Fig. 2c and Fig. 2d), but the number of water bridges was doubled for phosphorylated Tau – 646,827, compared to 321,546 for unmodified Tau. Interestingly, the number of individual water molecules mediating these bridges was practically identical – 47,761 and 47,529, respectively, out of around 48,000 water molecules in the systems. This means that nearly every water molecule in the unit cell becomes involved in interchain bridging interactions at least once during the molecular dynamics simulation.

Comparison between the spermine-containing systems to their spermine-free counterparts (Fig. 2e-h) shows that the behavior of the unmodified system is practically unperturbed upon addition of polyamine – in the spermine-free system, there are slightly more Na^+^-mediated bridging interactions (compare the yellow “curve” in Fig. 2e to the one in Fig. 2a; notice that it appears as yellow dots near the X axis in Fig. 2e and is barely visible in Fig. 2a), with the proportion of longer-lived bridges slightly increasing (compare the yellow curve in Fig. 2g to the one in Fig. 2c); the number and distribution of water-mediated bridges is practically unchanged (compare the blue curves in Fig. 2e to Fig. 2a and Fig. 2g to Fig. 2c). The difference, however, is much more dramatic for Na^+^ in between the phosphorylated systems, with the number of sodium-mediated bridges per frame increasing 8-fold in the spermine-free system; water-mediated bridges are only slightly affected (compare the yellow and blue curves in Fig. 2f to Fig. 2b). This confirms that spermine primarily competes with Na^+^ rather than water or Cl^−^ for protein binding sites.

### Tau phosphorylation greatly reduces spermine mobility

**Figure 3:**
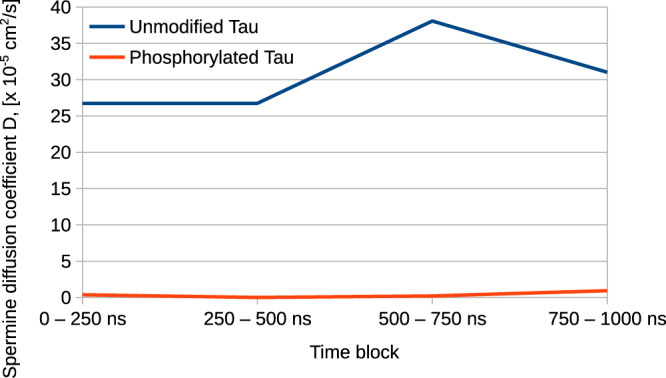
Spermine diffusivity in the Tau systems. Spermine diffusion coefficient calculated from the four time blocks (0–250, 250–500, 500–750, and 750–1000 ns) in the unmodified and phosphorylated Tau systems.

From the molecular dynamics trajectories, we also computed the diffusion coefficient (D) for spermine with unmodified and phosphorylated Tau. It was found that spermine has a nearly 100-fold greater diffusion coefficient with unmodified Tau versus the phosphorylated variant (D = 32 × 10^−5^ cm^2^/s for unphosphorylated Tau versus D = 0.29 × 10^−5^ cm^2^/s for phosphorylated Tau, as computed from the entire 1 μs molecular dynamics simulations). Examining the diffusion coefficients calculated from the four time blocks (Fig. 3) offers further detail on the behavior of the systems and confirms that the diffusive mobility of spermine is between 1 and 2 orders of magnitude smaller with phosphorylated Tau than with unmodified Tau.

**Figure 4:**
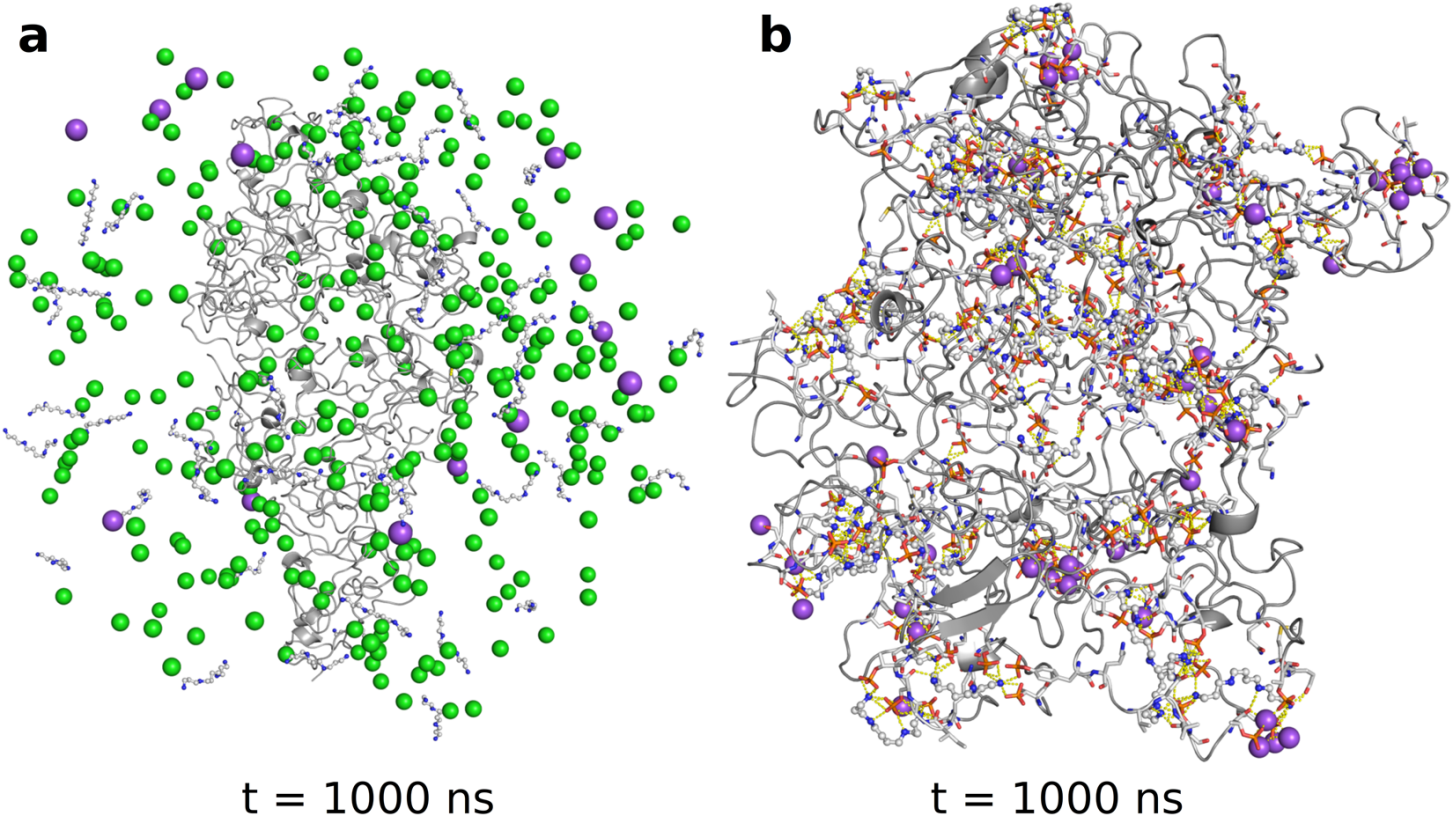
The Tau – spermine systems at the end of dynamics. (**a**) The unmodified Tau – spermine system at the end of dynamics. The backbone of the 8 Tau chains is shown in cartoon representation colored in gray. Na^+^ are shown as magenta spheres; Cl^−^ are shown as green spheres; spermine molecules are shown in ball-and-stick representation with nitrogen atoms in blue and carbon atoms in white. (**b**) A more zoomed-in view of the phosphorylated Tau – spermine system at the end of dynamics. Only ions within 3 Å of protein heavy atoms and making polar contacts are shown; the representation scheme for Na^+^ and spermine is as in. (**a**) The backbone of the 8 Tau chains is shown in cartoon representation colored in gray. Also shown in stick representation are protein residues making polar contacts with spermine and Na^+^ with oxygen atoms in red, carbon in white, nitrogen in blue, and phosphorus in orange; polar contacts are shown as yellow dotted lines.

Computing the diffusion coefficients of Na^+^ directly from the molecular dynamics trajectories and comparing them across systems is less reliable because the two systems have a different number of Na ions required for a net charge of zero (150 vs 14 for the phosphorylated and unmodified system, respectively). In particular, the small number of Na^**+**^ in the unmodified Tau system is likely insufficient to reliably calculate D. Nevertheless, the RDF curves and the bridging interactions analysis help one glean the behavior of Na^+^ around the protein. From Figs. 1 and 2, we see that Na^+^ becomes localized around the phosphoprotein with bridging interactions becoming longer-lived, implying that ion displacement due to random motion is reduced compared to native Tau. Thus, a clear picture of spermine and Na^+^ being preferentially positioned around the phosphorylated proteins, with diffusive motions slowed by favorable interactions with the macromolecules, begins to emerge. In stark contrast, with unmodified Tau, these moieties prefer to position themselves away from the protein chains, in bulk solvent, where they move freely, unaffected by the macromolecules. This is illustrated in Fig. 4 where at the end of molecular dynamics, no spermine- or ion-mediated bridging interactions are present in the unmodified system, with spermine and ions positioned away from the 8 protein chains. Conversely, at the end of dynamics in the phosphorylated system, Na^+^ and spermine are positioned around the protein, particularly around phosphate groups and acidic residues, making extensive salt bridges and salt-linked triads (an interaction where two salt bridges share a common residue). Moreover, visualizing the molecular dynamics trajectories shows that the described patterns for the two systems occur early on into the production dynamics step and persist until the end of the simulations.

### Spermine has much greater affinity for hyperphosphorylated Tau than the unmodified variant

**Figure 5:**
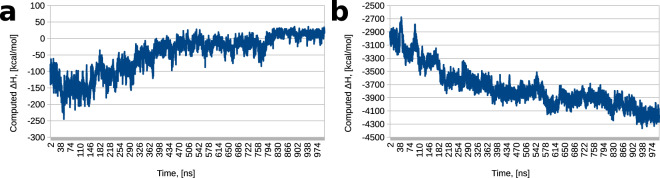
Enthalpies of Tau – spermine interaction. (**a**) Computed enthalpy of interaction between unmodified Tau and spermine over the course of production dynamics. (**b**) Computed enthalpy of interaction between phosphorylated Tau and spermine over the course of production dynamics. Note that the Y scales are different in the two panels.

**Figure 6:**
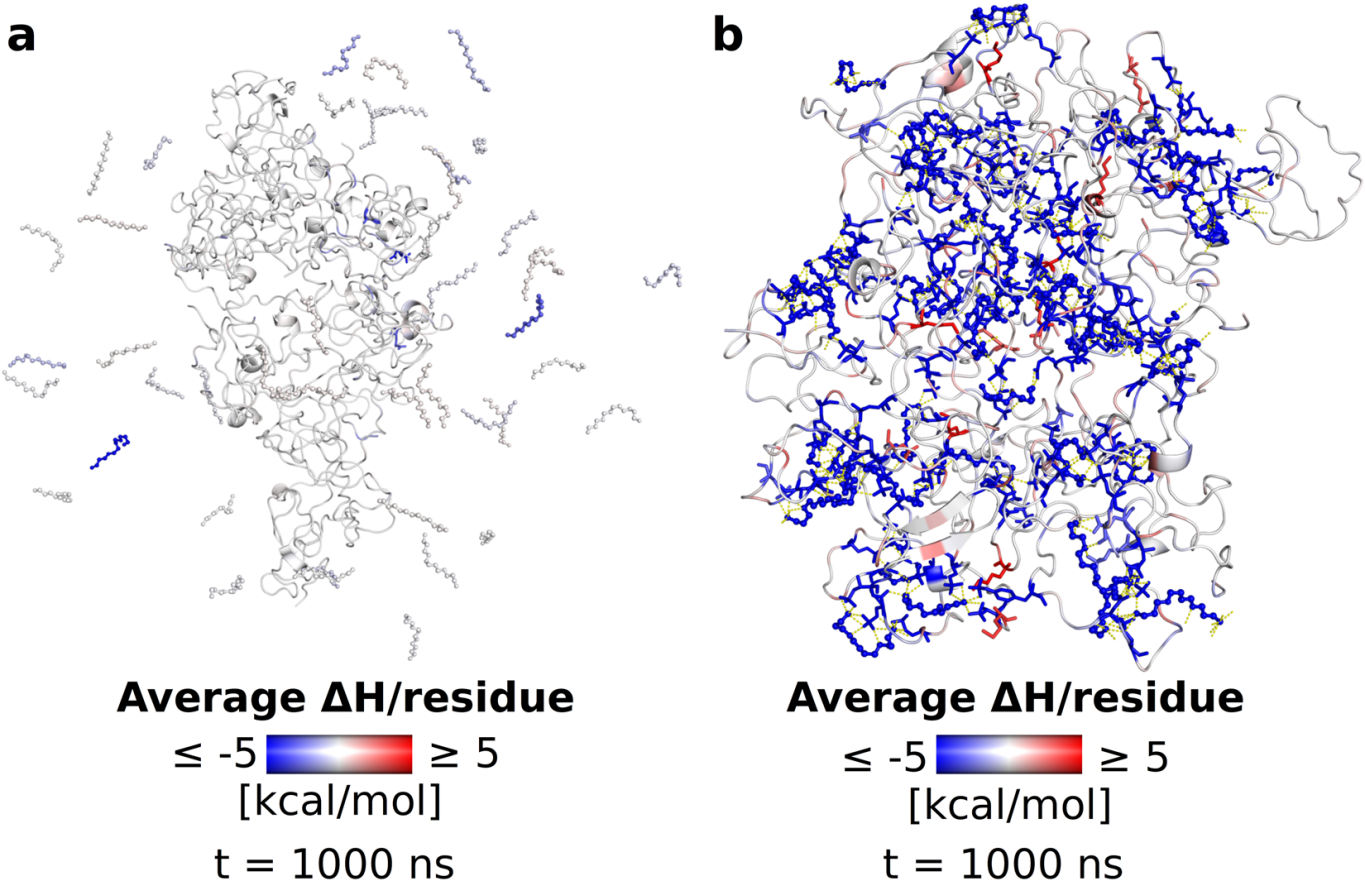
The Tau – spermine systems at the end of dynamics. (**a**) The unmodified Tau – spermine system at the end of dynamics. The backbone of the 8 Tau chains is shown in cartoon representation, spermine is in ball-and-stick representation. Tau and spermine are colored by per-residue energies of interaction calculated from the entire trajectory; blue indicates a favorable contribution to binding, red indicates an unfavorable contribution. Protein residues with contributions below −3 kcal/mol or above 3 kcal/mol are also shown in stick representation. Note that the frame in the image is the same as in Fig. 4a. (**b**) The phosphorylated Tau – spermine system at the end of dynamics. The backbone of the 8 Tau chains is shown in cartoon representation, spermine is in ball-and-stick representation. Tau and spermine are colored by per-residue energies of interaction calculated from the entire trajectory; blue indicates a favorable contribution to binding, red indicates an unfavorable contribution. Protein residues with contributions below −3 kcal/mol or above 3 kcal/mol are also shown in stick representation; polar contacts involving spermine are shown as yellow dotted lines. Note that the frame in the image is the same as in Fig. 4b.

In order to obtain a more quantitative assessment of the affinity between spermine and the two Tau variants, we performed molecular mechanics – generalized Born (MM-GBSA^35^) calculations, treating the 8 Tau chains as the “receptor” and the 44 spermines as the “ligand.” We also enabled per-residue energy decomposition during the MM-GBSA calculations, producing an assessment of each residue’s contribution to binding – favorable, unfavorable, or indifferent. Our results demonstrate that spermine has a much greater affinity for phosphorylated than unphosphorylated Tau. In the latter case, the energy of interaction between the 8 protein chains and the 44 polycations, presented here by the enthalpy of interaction (ΔH), plateaus around 0 near the end of the simulation (Fig. 5a), where spermine becomes removed from the protein, suggesting that it is energetically more favorable for spermine to interact with solvent than unmodified Tau. In stark contrast, spermine has great affinity for phosphorylated Tau – the ΔH values are large in magnitude and negative in sign, meaning that this is a highly favorable interaction (Fig. 5b; note that the Y scales are different in the two panels). Examination of the per-residue decompositions reveals that from the protein side, phosphorylated residues are the greatest contributors to Tau – spermine binding (Fig. 6). Overall, our RDF, bridging interactions, and MM-GBSA calculations and analyses convey a clear picture of phosphorylated Tau interacting strongly with the cellular polyamine spermine through its posttranslationally modified residues^36,37^ and unmodified Tau having low affinity for spermine. The two systems exhibit drastically different behavior despite having the same starting conformations.

## Discussion

Our molecular dynamics simulations and analysis provide detailed, atomic-resolution data on physiologically relevant Tau systems involving a previously overlooked but critical component of the neuronal cytosol – polyamines. We provide compelling computational evidence that phosphorylated Tau has high affinity for spermine. This is an important point to highlight because a higher affinity for each other than the remaining components of the environment would drive spermine-mediated phase separation^38^. While the computed energy of interaction between phosphorylated Tau and spermine is likely to be somewhat overly favorable because it does not explicitly take into account the loss in translational and rotational entropy^39–41^ due to the reduction in spermine mobility, it nevertheless provides a reliable estimate of the trend in affinity shift for polyamines upon Tau phosphorylation. To the best of our knowledge, we are the first to report on the possibility and the importance of Tau condensation by cellular polyamines.

The next point that warrants consideration is the concentrations of the components in our model systems. Due to the limitations of modern computing, we could simulate only 8 Tau chains in a volume of water of around 1,600,000 Å^3^ (~1.6 × 10^6^ Å^3^ or 1.6 × 10^−21^ L). This corresponds to a Tau concentration of around 8.2–8.3 mM, which is 82–83 times higher than the 100 micromoles used in the experiment^20^. However, the 100 μM value refers to the overall Tau concentration in the *in vitro* experiment, not the net concentration in the individual phases that were shown to form. It has been shown that in the case of proteinaceous liquid-liquid phase separation, the protein-rich phase can become enriched with protein nearly 100-fold compared to the protein-depleted phase^42,43^, implying that our system in reality is likely to be somewhat less concentrated than *in vitro*, and likel*y in vivo*, 4R-Tau droplets and fibrils. With regard to spermine, the concentration in our simulations is around 45 mM. Again, this is tens of times higher than the overall cellular concentration of ~1 mM; other cellular polyamines have even higher physiological concentrations^44^. However, this concentration of spermine corresponds to a neutralizing amount of charge for hyperphosphorylated Tau and is thus likely to be rather close to the local spermine concentration in a Tau-rich droplet.

It is noteworthy that even on the fairly limited timescale of our simulations (compared to experimental or *in vivo* conditions), we observed β-sheet formation by antiparallel β-strands; the astute reader will have noticed the antiparallel β-sheet in the lower parts of Figs. 4b and 6b. Although our starting structure contained no β-sheets or strands, several antiparallel sheets formed early on into the simulations and persisted throughout the molecular dynamics trajectories. It is conceivable, then, that the increased Tau concentration inside such liquid droplets leads to an increase in the local concentration of β-prone stretches of sequence (for example, VQIINK, which forms part of the upper strand in Figs. 4b and 6b) and serves as a primary nucleation center^38^ for the formation and growth of β-rich structures, transforming the innocuous liquid phase into solid-like, cytotoxic fibrils. This mechanism has been conjenctured before, with turbidity measurements, NMR and circular dichroism experiments, and differential interference contrast (DIC) microscopy^20^ providing low-resolution evidence to support it. We now describe atomic-, 100 picosecond-resolution data, which offers strong evidence in support of this mechanism.

While simulations alone cannot provide a definitive answer whether or not polyamines condense hyperphosphorylated Tau, our computational work shows that this is a plausible mechanism, warranting further experimental investigation. Indeed, only experiments can conclusively confirm or disprove our hypothesis. In particular, it is necessary to first establish the *in vitro* behavior of phosphorylated Tau in the presence of spermine and spermindine and to obtain a phase diagram of different tricomponent mixtures of Tau/phosphorylated Tau/polyamine near physiological concentrations and at core body temperature, closely mimicking *in vivo* conditions. Indeed, neurons are likely to contain a complex mixture of modified and unmodified forms of Tau, as opposed to the single-form Tau *in vitro* experiments often performed.

A key finding that emerges from the present analysis is the fact that phosphorylated^45^, i.e. posttranslationally modified, amino acids are the main residues responsible for the increase in affinity in Tau – spermine interactions. This becomes evident by noticing the clustering of spermine around these residues (see Fig. 4b) and the significant increase in residence time of spermine around them, elicited by the great number of hydrogen bonds and inter- and intrachain salt-linked triads. Bridging interactions and salt-linked triads, in particular, have been shown to be large contributors to affinity in intermolecular association^46,47^. Accordingly, the increase in phosphorylated Tau – spermine affinity is also reflected and becomes apparent in the per-residue decompositions (Fig. 6).

Notably, modulating affinity through reversible posttranslational modifications affords a mechanism for controlling the phase behavior of key cellular components and segregating them from and reintroducing them to the surrounding medium as needed. Indeed, it has been shown that dephosphorylaion of hyperphosphorylated Tau dissociates neurofibrillary tangles from AD brain samples and restores its biological activity, and that dephosphorylation of AD cytosolic hyperphosphorylated Tau inhibits its ability to selfaggregate^48^. It seems unlikely that RNA or heparin binding to Tau is the primary etiologic mechanism in Alzheimer’s disease for several reasons. First, pathology is strongly associated with the appearance of hyperphosphorylated Tau, upon which microtubules disassemble and Tau forms solid-like, insoluble filaments^49,50^. Inducing physiologically significant levels of condensation of hyperphosphorylated Tau via RNA or heparin *in vivo* is unlikely because of electrostatic considerations as well as the scarcity of heparin and long-lived, free RNAs inside the cytosol, even when accounting for continued RNA synthesis and replenishment. While electrostatic considerations would favor heparin- and RNA-induced condensation with unmodified Tau, and indeed this has been observed *in vitro* for heparin^20^, the scarcity factor is again a major limitation. Moreover, were physiological levels of glycosaminoglycans and/or RNA in neurons sufficient to trigger Tau tangle formation, AD prevalence or, at the very least, neurofibrilary tangle prevalence, would be much higher, likely being nearly universal in adults. Rather, our work points to phosphorylation-driven charge inversion and spermine-mediated Tau condensation followed by subsequent formation of β-rich, insoluble fibrils. Indeed, polyamine-mediated condensation followed by β-rich fibrillization has been shown for α-synuclein – the main protein component of Lewy bodies associated with Parkinson’s disease and dementia^51^. It was shown that physiological and even lower than physiological concentrations of the cellular polyamines spermine, spermindine, and putrescine accelerate the transition of the unstructured, negative-charge rich protein α-synuclein to β-rich, insoluble fibrils.

The localization of Na^+^ around phosphorylated Tau and the increase in residence time of Na^+^ around phosphorylated Tau point to the possibility of metal ions contributing to condensation. Indeed, it was observed experimentally^20^ that condensation and fibrillization are favored between 0 and 150 mM NaCl but are greatly reduced past concentrations beyond 200 mM, presumably because of screening effects. It has been shown that polyvalent ions are much more effective condensing agents than monovalent ones, with condensing potential increasing greatly with ion charge^52^. This holds for metal ions, as well as polyamines^32,51^. Thus, one might expect physiological amounts of intracellular Mg^2+^ and Zn^2+^, among other ions, to contribute to Tau condensation and fibrillization. Similarly, Uversky *et al*. have shown that metal polycations of high charge density aggregate and fibrillize α-synuclein^53^. Moreover, it has been shown that AD is also associated with Tau lysine acetylation, along with phosphorylation^54^. Indeed, it was demonstrated that acetylation contributes to Tau aggregation^55,56^. This provides indirect evidence for our hypothesis, as lysine acetylation contributes to Tau charge suppression and inversion and also provides an additional hydrogen bond acceptor site (the CO group) and steric bulk (from the entire CH_3_CO group) for every acetyl group added, facilitating aggregation and subsequent fibrillization.

It is now known that Tau is subject to modification by multiple kinases, phosphatases, acetylases, deacetylases, and proteases, among many other enzymes which modify its structure. Different kinases have different specificity and efficiency. For example, microtubule-associated protein/microtubule affinity-regulating kinase 2 (MARK2) completely phosphorylates S579, S641, and S673 and phosphorylates S610, S622, and S669 with an efficiency between 10 and 20%^20^ (the numbering corresponds to the canonical Uniprot^57^ sequence); S602 and S622 are phosphorylated by PHK^58^, and so on. The list of known Tau posttranslational modifications already has almost 100 entries and can only grow further as more experimental data become available. Moreover, mutations of serines to glutamic acid residues that emulate the effect of phosphorylation have been shown to make Tau significantly more prone to aggregation and fibrillization^18^. We expect there to be a critical amount of phosphorylation which triggers Tau aggregation and fibrillization, with different combinations of phosphorylation sites and mutations likely exhibiting different propensities to aggregate and fibrillize. Examining such combinations is computationally unfeasible but experimentally quite tractable.

We base our proposed mechanism of Tau fibril formation on a detailed, atomistic understanding of the behavior of the physiologically relevant model systems, grounded in cellular biology. It is also in this detailed understanding that our proposed etiologic mechanism for Alzheimer’s disease is rooted. If, as we propose, hypeprhosphorylated (potentially also acetylated) Tau condenses and forms insoluble, cytotoxic fibrils through interactions with cellular polyamines (and potentially also cytosolic metal ions and/or other cations), then there should exist a detectable pattern of kinase and/or phosphatase dysfunction/mutation in AD patients. It is also possible that dysfunction in neuronal acetylases/deacetylases and/or ion transport/homeostasis are contributing factors. Given that the human proteome encodes over 500 putative kinases alone^59^, this might seem like a daunting task. However, using the tools modern bioinformatics and computing provide, sequencing^60^ of patient samples and healthy controls makes searching for this bioinformatics signature quite approachable. Moreover, one should narrow down the search to enzymes and proteins expressed in neurons; ones not expressed in neurons likely have no relation to Tau fibrillization in AD and should be excluded from consideration as potential suspects. Finally, much like in AD, Tau phosphorylation and aggregation have been noted to occur in Down syndrome^61–64^ (trisomy 21), with brain changes of aged individuals with Down syndrome nearly identical to those of AD patients, consisting of both senile plaques and neurofibrillary tangles composed of Aβ and phosphorylated Tau, respectively^65^. This suggests that the molecular drivers of Alzheimer’s disease are encoded in chromosome 21, further narrowing the search space. In the case of glial fibrillary tangles composed of hyperphosphorylated Tau^66–68^, the search should focus on targets expressed in astrocytes.

We expect that if our proposed mechanism is confirmed, different “subtypes” of Alzheimer’s disease will be identified, corresponding to different patterns of hyperphospohorylation, which, in turn, would likely correspond to different fibrils with different properties. Should a distinct molecular target or set of targets in AD emerge, the condition is likely to become much more tractable, even if there is the problem of selectively targeting one protein while avoiding any disruption to a large set of highly similar paralogs^46,69,70^.

Nevertheless, one should not forget that Alzheimer’s is a complex, multifaceted disease where multiple factors likely make varying contributions to etiology and pathology. Many gaps in our understanding of the key mechanisms and players in disease etiology still remain. For example, it has been argued that Tau oligomers, rather than fibrils, are the primary neurotoxic agents^71^. Others have pointed out that spermine and the broader category of cellular polyamines are free radical scavengers^72^ and that they serve a neuroprotective role upon heavy metal exposure^53^. By condensing α-synuclein^51^ and, as we propose, Tau, they inadvertently and paradoxically trigger neurodegeneration. Both of these mechanisms are consistent with our hypothesis, as is the possibility that Tau fibrils are the primary neurotoxic agents, rather than the oligomers^73^. Moreover, despite the multiple clinical trial failures, the amyloid hypothesis still has proponents. Indeed, evidence showing that high doses of aducanumab slow the rate of Alzheimer’s progression has been put forth, prompting the developer to attempt to gain regulatory approval. This implies that targeting amyloid beta may confer some benefit, even if Aβ is not the main etiologic driver of the disease. Similarly to Tau, there is uncertainty whether the oligomers or the fibrils are the primary neurotoxic forms of Aβ^9^. Researchers have further pointed out the need to control cerebral inflammation in AD that is triggered by Tau and Aβ and may persist even after the Tau and Aβ deposits have been cleared^74^. Industry experts have also expressed the opinion that many of the previous clinical trials may have failed because treatment was administered too late, thereby masking the beneficial effects of drugs that could have potentially been successful^4,7,9^. Crucially, better study design in future clinical trials is needed in order to detect weak signals and reliably identify clear results in such a complex neurological condition^7,8^.

## Methods

### System preparation

For unmodified Tau, we used the exact sequence used in the experimental measurements (the sequence is referred to as “K18” in that paper^20^). We used the first Tau chain from the 2MZ7 NMR structure^75^ as a template, adding the missing N- and C-terminal residues with MODELLER 9.20^76^. As this sequence is intrinsically disordered, we did not introduce any secondary structure constraints into the modelling step; we only opted for a more compact structure to minimize the number of water molecules needed to solvate the system in order to reduce the computational burden during molecular dynamics. We also selected a structure with a favorable Ramachandran plot, i.e. among the different structures produced by MODELLER, we chose one with minimal backbone strain^77^. The Tau chain was then energy minimized with FoldX 5.0^78^. We constructed the model systems by randomly placing 8 Tau chains inside a unit cell and again minimizing with FoldX. For the spermine-containing systems, we randomly added 44 spermine molecules. Although other cellular polyamines have higher concentrations than spermine, we chose this polyamine as it has the highest charge and the highest condensing potential^32,51^, thereby likely making its effects more pronounced and detectable by molecular dynamics simulations. To avoid ambiguity in constructing the phosphorylated Tau systems and to make the effects of phosphorylation detectable by MD, we opted to phosphorylate all serine (12), threonine (4), and tyrosine (1) residues in the sequence, giving a single chain a net charge of −22. Having 8 such chains in the system, which was the computationally feasible maximum, required 44 spermine molecules for charge neutrality. Protein chains were protonated and solvated in a truncated octahedral box with TIP3P water^79^ with *tleap* from the Amber18 package^80^ with a minimal wall distance of 13 Å, for a total system size of around 160,000 atoms. 0.150 M of NaCl with Joung and Cheatham parameters were added to approximate a physiological salt concentration while maintaining overall charge neutrality. Amber-compatible parameters for the phosphorylated residues were obtained from Forcefield_PTM^81^ hosted at Princeton University; parameters for spermine were obtained using the general Amber force field^82^ (GAFF 2.11) with AM1-BCC charges^83^ using *antechamber*.

### Molecular dynamics simulations

The solvated systems were subjected to 5,000 steps of energy minimization with 3 kcal*mol^−1^ *Å^−2^ harmonic restraints on solute heavy atoms, followed by heating from 0 to 300 K over a period of 1 ns at constant volume with identical restraints, followed by 1 ns of constant pressure density equilibration with restraints. The systems were then equilibrated for 100 ns without any restraints and then simulated for 1,000 ns (1 μs) of production dynamics under constant temperature (300 K) and pressure (1 bar), maintained with the Langevin thermostat^84^ and Berendsen barostat^85^, respectively. Collision frequencies for temperature coupling were set to 2 ps^−1^; the pressure relaxation time was set to 2 ps using isotropic position scaling. The systems were simulated with the ff14SB^86^ force field under periodic boundary conditions. A 12.0 Å cutoff was used for both van der Waals and electrostatic interactions; long-range electrostatics beyond the real-space cutoff were evaluated with the particle-mesh Ewald (PME) scheme^87^. During heating, density equilibration, preproduction, and production dynamics, covalent bonds involving hydrogen were constrained using the SHAKE algorithm^88^, allowing for a 2 fs time step; only during energy minimization bonds to hydrogen were not constrained. During production dynamics, frames were saved every 100 ps (0.1 ns) for a total of 10,000 per trajectory, to be used in subsequent analysis.

### Identification of bridging interactions, residence time analysis, and MM-GBSA calculations

For this step, we used the pipeline we have developed and described in a previous publication^47^. Briefly, for each frame from the production dynamics trajectories, bridging water molecules, spermine molecules, and ions were identified with the *hbond* command for *cpptraj V4.24.0*^89^ using the *nointramol* keyword, which excludes intramolecular bridging molecules and ions from consideration. The distance cutoff for *hbond* was 3 Å (donor to acceptor heavy atoms), the angle cutoff was 135 degrees. The number of bridging molecules and ions was calculated, as were the total number of bridging interactions formed during the 1,000 ns simulations, and the lifetimes of the bridging molecules. Herein, we define a bridging molecule or ion as one that is simultaneously hydrogen bonded to two or more Tau chains in at least one of the 10,000 frames per production dynamics run. When counting the number of bridges in a trajectory, we defined each bridge as a single, uninterrupted interaction. For example, if a given spermine molecule is involved in bridging interactions in frames 511, 600, 650, 651, 700, 701, 702, and 703, we record four separate bridges with lifetimes of 0.1, 0.1, 0.2, and 0.4 ns, respectively. A given molecule or ion can be involved in multiple bridging interactions; hence, the number of bridges in a molecular dynamics simulation is typically much larger than the number of bridging molecules. For example, a given spermine molecule may become involved in a single, long-residence time bridging interaction where it remains located between two Tau chains for several nanoseconds without interruption. Then, it can leave this location and return to it multiple times for short periods of time. Hence, we record one bridging spermine molecule and several bridging interactions – one long-lived and multiple short-lived bridges. In the present report, we use the phrases “lifetime of a bridging interaction” and “residence time of a molecule/ion” interchangeably. We generated the radial distribution functions for water and spermine heavy atoms, Na^+^, and Cl^−^ around protein heavy atoms with the Gromacs *rdf* tool; diffusion coefficients were computed with *cpptraj*.

The enthalpy of interaction between the protein chains and spermine (ΔH) was computed with the MMPBSA.py tool^90^, part of the Amber18 package. Per-residue energy decomposition was performed for every individual frame by adding 1–4 energy terms to internal energy terms; the energies for every residue were averaged over the 10,000 frames of production dynamics to produce an assessment of each individual residue’s contribution to binding over the course of the entire trajectories. More details on the theory and parameters of the MM-GBSA calculations and our pipeline can be found in our previous publication^47^.

## Data Availability

All relevant data are available from the corresponding author upon reasonable request.

## Code availability

All relevant code is available from the corresponding author upon reasonable request.
